# Phenolics Profile and Antioxidant Activity of Special Beers

**DOI:** 10.3390/molecules25112466

**Published:** 2020-05-26

**Authors:** Mirella Nardini, Maria Stella Foddai

**Affiliations:** CREA, Research Centre for Food and Nutrition, via Ardeatina 546, 00178 Rome, Italy; mariastella.foddai@crea.gov.it

**Keywords:** beer, polyphenols, antioxidant activity, walnut, chestnut, green tea, coffee, cocoa, honey, licorice

## Abstract

The antioxidant activity and polyphenols content of beer associated with its low alcohol content are relevant factors for an evaluation of the nutritional quality of beer. To investigate the effect of adding foods on the nutritional quality of beer, seven special beers that were commercially available and produced adding natural foods (walnut, chestnut, cocoa, honey, green tea, coffee, and licorice) during the fermentation process were analyzed for their polyphenols and flavonoids contents, phenolics profile, and antioxidant activity. The results obtained showed that most of the special beers under study possessed antioxidant activity, as well as total polyphenols and flavonoids contents notably higher as compared with the five conventional beers analyzed. The highest polyphenols and flavonoids contents were exhibited in cocoa, walnut, chestnut, and licorice beers, followed by coffee, honey, and green tea beers. Antioxidant activity decreased in the order walnut, cocoa, chestnut, licorice, coffee, honey, and green tea. Most special beers were enriched in catechin, epicatechin, rutin, myricetin, quercetin, and resveratrol. The content of phenolic acids, especially ferulic, *p*-coumaric, syringic, and sinapic acids was generally higher in special beers as compared with conventional beers. Our findings showed that the addition of natural foods during the fermentation process remarkably increased antioxidant activity of beer and qualitatively and quantitatively improved its phenolics profile.

## 1. Introduction

Oxidative stress is involved in the pathology of several human diseases, such as atherosclerosis, diabetes, neurodegenerative diseases, ageing, and cancer [1]. Dietary antioxidants can counteract the negative effects of oxidative stress. Polyphenols are the most abundant dietary antioxidants, due to their presence in all fruits and vegetables [1]. Polyphenol intake can be several hundreds of milligrams per day, up to 1 g/day, depending on dietary habits [2] and, in particular, in wine, coffee, beer, chocolate, and tea consumption; and it largely exceeds that of other antioxidants, such as vitamin E, vitamin C, and β-carotene [3]. Among polyphenols, phenolic acids account for about one-third of the total intake, while flavonoids account for the remaining two-thirds of the total intake [2]. Epidemiological studies have suggested associations between long-term consumption of polyphenols-rich foods and prevention of oxidative stress-related diseases such as cancer, cardiovascular diseases, diabetes, inflammation, and degenerative diseases [1,4,5,6].

Beer is one of the most popular alcoholic beverages consumed in large amounts all over the world, being a source of carbohydrates, amino acids, minerals, vitamins, and polyphenols. About 30% of beer polyphenols originate from hops, while the remaining 70% come from malt [7,8]. Moreover, hops provide compounds which become bitter acids (humulones) during the beer fermentation process [8]. The antioxidant activity and polyphenols content of beer associated with its low alcohol content are relevant factors in evaluating the nutritional quality of beer. Moderate beer drinking has been reported to increase plasma antioxidant and anticoagulant activities, to positively affect plasma lipid levels, and to exert protective effects on cardiovascular risk in humans [9,10,11,12].

In addition to the most familiar products, special beers produced with the addition of fruits, spices, or natural food during the fermentation process, have been becoming very popular throughout the world, responding to requests for new gustatory, olfactory, and visual stimuli from consumers. During re-fermentation and maturation of special beers, flavors and bioactive compounds, such as carotenoids and polyphenols, are extracted from fruits, spices, and natural food added to beer. Recently, the addition of fruits during the fermentation process has been reported to significantly increase the content of bioactive compounds and the antioxidant activity of beer [13]. Despite many studies describing the raw materials and the effects of technological processes, little is known about the healthy compounds and nutritional quality of commercially available beers [14,15,16].

In order to investigate the effect of several food additions on the nutritional quality of beer, we investigated total polyphenols and flavonoids contents, phenolics profile, and antioxidant activity of seven special beers produced with the addition of walnut, chestnut, cocoa, green tea, coffee, honey, or licorice during the fermentation process and compared our results with five conventional beers.

## 2. Results

### 2.1. Beers’ Characterization

Conventional and special beers were examined in this study. The special beers were produced by the addition of the following different foods: walnut (*Juglans Regia* L., WALN), chestnut (*Castanea Sativa* L., CHES), green tea (*Camelia Sinensis* L., GTEA), coffee (*Coffea Arabica and Coffea Robusta* L., COFF), cocoa (*Theobroma Cacao* L., COCO), honey (*Wildflower honey*, HONE) and licorice (*Glycyrrhiza Glabra* L., LIQU), as shown in Table 1. The amount of the foods added varied in the different special beers from 2 to 62.5 g/L of beer.

The characteristics of special and conventional beers are summarized in Table 2. All special beers were produced in Italy and were ale style beer (high fermentation beer), except one (CHES beer) which was a lager style beer (low fermentation beer). All conventional beers were produced in Italy, except one (ALE 1) which was produced in Belgium. Three conventional beers were ale style beers and two conventional beers were lager style beers. The alcoholic strength was in the range 4.5%–9.0% and 4.6%–6.6% for special and conventional beers, respectively (Table 2). Among the special beers, licorice (LIQU) and chestnut (CHES) beers exhibited the highest alcohol content (9.0% and 8.0%, respectively), while the alcohol content of the remaining special beers was quite close to that of conventional beers. The pH was in the range 4.04–4.64 and 4.29–4.87 for special and conventional beers, respectively (Table 2). International Bitterness Unit (IBU) values were in the range 7–30 for special beers, with the highest value reported for walnut beer (WALN), and in the range 15–35 for conventional beers, with the highest value reported for ALE 3 beer (Table 2). European Brewery Convention (EBC) values, referring to the color intensity of beer, were in the range 5–110 for special beers, with the highest value reported for coffee (COFF) and cocoa (COCO) beers, and in the range 4–20 for conventional beers, with the highest value reported for ALE 3 beer (Table 2).

### 2.2. Total Polyphenols and Flavonoids Contents of Beers

Most special beers (six out of seven) showed total polyphenols content considerably and significantly (*p* < 0.05) higher (range 464–1026 mg/L of beer) as compared with that of the conventional beers (range 274–446 mg/L of beer) (Table 3). The highest polyphenols level was measured in cocoa (COCO) beer, followed by walnut (WALN), chestnut (CHES), licorice (LIQU), coffee (COFF), honey (HONE), and green tea (GTEA) beers. The polyphenols content of conventional beers was in the same order of that reported in our previous studies and in the literature [13,16,17,18].

Total flavonoids content of special beers was in the range 42–96 mg/L of beer. These values are somewhat higher as compared those measured in conventional beers (range 27–63 mg/L of beer) (Table 3). Among the special beers, the highest flavonoids content was measured in cocoa beer (COCO), followed by walnut (WALN), licorice (LIQU), chestnut (CHES) and coffee (COFF) beers, whereas honey (HONE) and green tea (GTEA) exhibited a total flavonoids content close to that of conventional beers.

**Figure 1:**
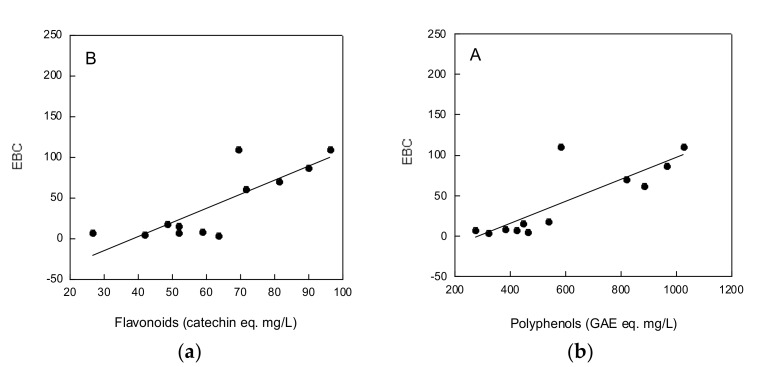
Relationship between beer EBC values and polyphenols (**a**) or flavonoids (**b**) contents. Data were analyzed for correlation by Student’s *t*-test.

A significant correlation was found between EBC values and total polyphenols (Figure 1a) or flavonoids (Figure 1b) contents (*p* < 0.001, R = 0.82878 and *p* < 0.005, R = 0.81706, respectively).

### 2.3. Beers Antioxidant Activity

The antioxidant activity measured with ferric reducing antioxidant power (FRAP) assay was considerably higher in special beers (FRAP range 3.6–10.2 mM Fe_2_SO_4_/L of beer) as compared with that of conventional beers (range 1.7–3.9 mM Fe_2_SO_4_/L of beer) (Table 3). In the same way, the antioxidant activity evaluated by 2,2′-azino-bis(3-ethylbenzothiazoline-6-sulfonic) acid (ABTS) radical cation decolorization assay showed higher values in special beers (range 2.4–5.2 mM Trolox/L of beer) as compared with those of conventional beers (range 1.5–2.6 mM Trolox/L of beer). The highest antioxidant activity was measured in walnut (WALN) beer, followed by cocoa (COCO), chestnut (CHES), licorice (LIQU), and coffee (COFF) beers. Honey (HONE) and green tea (GTEA) beers showed antioxidant activity values close to those measured in conventional beers (Table 3). The antioxidant activity values measured in conventional beers were consistent with our previous results and with data from the literature [10,13,16,19].

**Figure 2:**
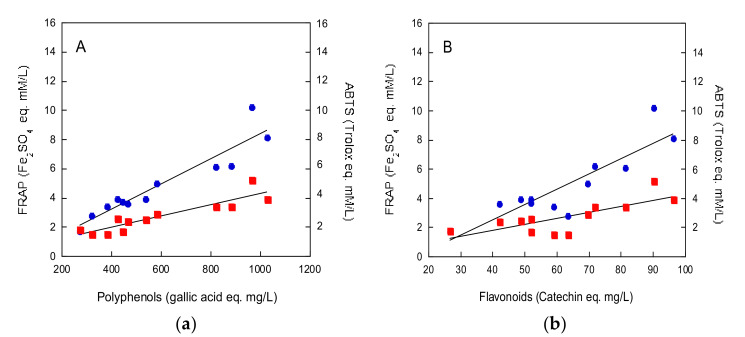
Relationship between beer antioxidant activity, measured by both FRAP (full circles) and ABTS (full squares) assays, and polyphenols (**a**) or flavonoids (**b**) contents. Data were analyzed for correlation by Student’s *t*-test.

As shown in Figure 2a, a strong correlation between total polyphenols content, measured by Folin–Ciocalteu assay, and antioxidant activity of beers, measured by both the FRAP and ABTS assays, was found (*r* = 0.93815, *p* < 0.0001 for FRAP assay and *r* = 0.90592, *p* < 0.0001 for ABTS assay). Furthermore, a strict correlation was observed between the total flavonoids content and the antioxidant activity of beers, measured by both the FRAP and ABTS methods (*r* = 0.87913, *p* < 0.0002 for FRAP assay and *r* = 0.75286, *p* < 0.005 for ABTS assay) (Figure 2b).

### 2.4. Phenolics Profile Analyses

Due to the role of polyphenols in determining beer quality, the hydroxycinnamic acid derivatives chlorogenic, vanillic, caffeic, *p*-coumaric, and ferulic acids; the hydroxybenzoic acid derivatives syringic and sinapic acids; the flavonoids catechin, epicatechin, rutin, myricetin, and quercetin; and the stilbene derivative resveratrol were measured by high-performance liquid chromatography (HPLC). As most phenolic acids are present in beer as esterified forms, we measured the level of both free and total (free plus conjugated forms) phenolic acids [16]. The content of single phenolic compounds, representative of the different classes of polyphenols, are shown in Table 4 and Table 5 for conventional and special beers, respectively.

As a basis for comparison, first, conventional beers were analyzed. The total phenolic acids content of conventional beers, obtained by alkaline hydrolysis, varied in the range 21.78–38.89 mg/L of beer (Table 4). Total ferulic acid was by far the most abundant phenolic acid in conventional beers, regardless of the beer style, ranging from 10.27 to 21.66 mg/L of beer, followed by caffeic (range 1.61–5.99 mg/L of beer), sinapic (range 2.19–4.80 mg/L of beer), vanillic (range 2.30–4.65 mg/L of beer), and *p*-coumaric (range 0.77–2.77 mg/L of beer) acids, whereas syringic acid exhibited the lowest concentration (range 0–0.71 mg/L of beer). Lager style beers (LAGE 1 and LAGE 2) showed the lowest caffeic, syringic, and *p*-coumaric acids content as compared with ale style beers. The total amount of each phenolic acid, measured after alkaline hydrolysis, was higher with respect to the content of the respective free form, indicating that phenolic acids were present in beer mainly as conjugated forms. Free and total phenolic acids contents of conventional beers was in the same order of magnitude as that reported in our previous studies [13,16,20]. Free phenolic acids content measured in conventional beer was also in agreement with other data from the literature [21,22,23,24,25], whereas total phenolic acids content is usually not routinely measured. Noteworthily, chlorogenic acid; the flavonoids catechin, epicatechin, rutin, myricetin, and quercetin; and the stilbene derivative resveratrol were undetectable in all conventional beers in our experimental conditions, regardless of the beer style (Table 4).

The phenolic profile of special beers is shown in Table 5. The content of single phenolic acids differs considerably among the different special beers. Total phenolic acids content obtained after alkaline hydrolysis varied in the range 20.54–45.45 mg/L of beer, with chestnut (CHES) beer exhibiting the highest value, followed by cocoa (COCO), licorice (LIQU), coffee (COFF), honey (HONE), green tea (GTEA), and walnut (WALN) beers. Ferulic acid was by far the most abundant phenolic acid in all special beers, while syringic acid showed the lowest values, as found in conventional beers. In detail, total ferulic and vanillic acids varied in the ranges 8.22–27.55 and 2.03–5.09 mb/L of beer, respectively, with the highest value measured in chestnut (CHES) beer. Total caffeic acid content ranged from 1.48 to 9.20 mg/L of beer, with the highest value measured in coffee (COFF) beer and the lowest value in green tea (GTEA) beer. The total *p*-coumaric content ranged from 1.75 to 4.32 mg/L of beer, with the highest content in walnut (WALN) beer. The total sinapic acid content of special beers varied in the range 2.52–6.73 mg/L of beer, the highest values found in honey (HONE) beer, followed by licorice (LIQU), cocoa (COCO), chestnut (CHES), green tea (GTEA), walnut (WALN), and coffee (COFF) beers (Table 5). The total syringic acid content ranged between 0.67–1.42 mg/L of beer, with the highest value found in cocoa (COCO) beer, while it was undetectable in walnut (WALN) and coffee (COFF) beers, in our experimental conditions. The total amount of each phenolic acid measured after alkaline hydrolysis was higher with respect to the content of the respective free form, also indicating that, in the special beers, phenolic acids were mainly present as conjugated forms. Noteworthily, among the special beers, chlorogenic acid was detected only in coffee (COFF) beer.

Unlike conventional beers, the special beers under study exhibited detectable levels of the flavonoids catechin, epicatechin, rutin, myricetin, quercetin, as well as the stilbene resveratrol. The flavonoids epicatechin, myricetin, quercetin, as well as the stilbene resveratrol were present in almost every special beer under study, whereas the flavonoids catechin and rutin were detectable in three out of the seven special beers, in our experimental conditions (Table 5). The epicatechin content varied in the range 0.94–3.68 mg/L of beer, with the highest values measured in chestnut (CHES) and green tea (GTEA) beers, while only traces were found in licorice beer (LIQU). Myricetin and quercetin content varied in the range 0.39–8.82 and 0.54–6.55 mg/L of beer, respectively, with the highest myricetin value found in licorice (LIQU) beer and the highest quercetin level measured in walnut (WALN) beers, whereas only trace amount of both flavonoids were measured in chestnut (CHES) beer. Catechin was present in the range 2.98–4.65 mg/L of beer in chestnut (CHES), green tea (GTEA), and cocoa (COCO) beers, whereas only traces were detected in the remaining special beers. Rutin was detected in green tea (GTEA), honey (HONE), and licorice (LIQU) beers, ranging from 0.68 to 1.29 mg/L of beer (Table 5).

In regard to the stilbene derivative resveratrol, it was present in all special beers under study, although at a low level (range 0.20–0.35 mg/L of beer).

## 3. Discussion

The antioxidant activity and phenolics content of beer rely on the quantity and quality of starting material, as well as on the industrial brewing process. Beer exhibiting high phenolics content and high antioxidant activity display better quality, more stable flavor and aroma, foam stability, and longer shelf life as compared with beer with lower phenolics levels and weaker antioxidant properties [7,26,27,28,29].

In our study, total polyphenols and flavonoids contents of most special beers was remarkably higher as compared with conventional beers. In particular, the flavonoids catechin, rutin, myricetin, quercetin, as well as the stilbene, resveratrol, were undetectable under our experimental conditions, in all conventional beers analyzed.

Undoubtedly, beer color has an impact on beer taste and experience. The EBC values were remarkably higher in special beers as compared with conventional beers. The strong correlation found between EBC values and total polyphenols and flavonoids contents suggest a relevant contribution of plant food phenolics to special beer color, in addition to that of malt. A similar correlation between beer total polyphenols content and EBC values has been recently reported, which suggested that beer color is correlated to the total amount of phenolic compounds [30]. Instead, the IBU values gave similar bitterness values in both special and conventional beers.

Recently, the addition of fresh fruits during the fermentation process has been reported to increase antioxidant activity, total polyphenols and flavonoids contents, and to qualitatively and quantitatively improve the phenolics profile with respect to conventional beers [13]. In this study, the special beers produced with food addition during the fermentation step exhibited total polyphenols content and antioxidant activity even higher than those reported for fruit beers. Notably, the specific foods involved in the present study have been reported to contain high polyphenols levels and to possess strong antioxidant activity [31]. The strict correlation observed between antioxidant activity and total polyphenols and flavonoids contents suggest a central role of phenolics in the antioxidant properties of beers.

Our results showed that cocoa (COCO), walnut (WALN), chestnut (CHES), and licorice (LIQU) beers exhibited the higher polyphenols and flavonoids contents, followed by coffee (COFF), honey (HONE), and green tea (GTEA) beers. Antioxidant activity decreased in the order walnut (WALN), cocoa (COCO), chestnut (CHES), licorice (LIQU), coffee (COFF), honey (HONE), and green tea (GTEA) beers. The phenolic profile obtained by HPLC showed that most special beers are enriched in catechin, epicatechin, rutin, myricetin, quercetin, and resveratrol. Phenolic acids content, especially ferulic, *p*-coumaric, syringic, and sinapic acids, was generally higher in special beers as compared with the conventional beers.

Walnut beer (WALN) showed the highest antioxidant activity, measured by both FRAP and ABTS assays and high total flavonoids level. The HPLC analyses demonstrated the highest quercetin content among special beers, in addition to high levels of epicatechin and myricetin. In this regard, walnuts have been reported to contain many phytochemicals, including the highest known levels of phenolic antioxidants (phenolic acids, flavonoids, and tannins) with respect to other nut species [32,33,34].

Chestnut beer (CHES) exhibited the highest catechin, epicatechin, and resveratrol levels. Accordingly, chestnuts have been reported to contain high levels of catechin, and epicatechin, in addition to phenolic acids and tannins [35,36,37]. Moreover, chestnuts have been recognized as one of the richest foods with respect to polyphenols content, exhibiting very high antioxidant activity [31].

Among special beers, coffee (COFF) beer showed the lowest catechin, rutin, myricetin, quercetin, epicatechin, and resveratrol levels. However, coffee beer contained chlorogenic acid and the highest caffeic acid level among the special beers. Accordingly, both caffeic and chlorogenic acids have been reported to be present in high amounts in coffee [38,39] and are extracted from coffee during the fermentation process of coffee beer.

Cocoa and green tea are known to possess high polyphenols and flavonoids contents, especially catechin and epicatechin [31,40,41]. In agreement, high levels of catechin and epicatechin together with quercetin and myricetin were detected in both cocoa (COCO) and green tea (GTEA) beers, indicating, once again, that these compounds are extracted from cocoa and green tea during the fermentation process.

Despite the low amount of licorice added to beer (2 g/L of beer) during the fermentation process, licorice (LIQU) beer exhibited the highest myricetin content as compared with the other special beers together with high levels of quercetin, caffeic, *p*-coumaric, ferulic, and sinapic acids. Licorice has been reported to contain many bioactive compounds, particularly flavonoids, which are responsible for its yellow color [42,43]. Various biological activities have been associated with licorice extracts, particularly with its flavonoids and triterpenic saponins contents, such as antiviral, antimicrobial, antioxidant, anti-inflammatory, and anticancer effects [44,45].

Honey beer (HONE) showed the highest sinapic acid and rutin contents as compared with the other special beers, and high levels of mirycetin and quercetin. According to our results, the occurrence of caffeic acid, *p*-coumaric acid, ferulic acid, vanillic acid, sinapic acid, syringic acid, rutin, quercetin, myricetin, resveratrol, and epicatechin in honey has been reported by several studies [46,47]. Honey is one of the most renowned natural foods. Although its composition is extremely variable, depending on its botanical and geographical origins, the abundant presence of phenolic compounds, especially phenolic acids and flavonoids, and the antioxidant properties of honey have renewed interest toward this natural food.

In our previous study [16], we demonstrated that phenolic acids strongly contribute to the antioxidant activity of beer. Flavonoids have been reported to be free radical scavengers, metal chelators, and strong antioxidants [48,49]. Therefore, the enrichment in flavonoids observed in the special beers could account, at least in part, for the higher antioxidant activity measured in most of the special beers as compared with the conventional beers. The stilbene derivative resveratrol was also detected in the special beers, although at low levels, and it could contribute to the antioxidant activity of beers.

Antioxidant activity and polyphenols content of beer associated with its low alcohol content are relevant factors in determining the nutritional quality of beer. Total polyphenols content of conventional beer is quite low as compared with that of red wine. In fact, the total amount of polyphenols in red wine has been estimated to be in the range 2000–6000 mg/L of wine [50,51,52,53], whereas that of conventional beers has been reported to vary in the range 300–500 mg/L of beer for the most common beer styles [13,16]. Higher values (622 ± 77 and 875 ± 168 mg/L, respectively) have been reported only for abbey and bock beer styles [16]. However, the polyphenols content of conventional beers has been reported to be similar or even higher with respect to that reported for white wine (range 50–350 mg/L) [50,51,52]. Recently, a total polyphenols content of up to 770 mg/L of beer has been reported for fruit beers, produced through the addition of fresh fruits during the fermentation process [13]. The special beers examined in this study exhibited total polyphenols content in the range 464–1026 mg/L, even higher than that reported for fruit beers. These values are substantially higher as compared with those of the conventional beers, as well as compared with those of white wine. A similar trend could be observed for antioxidant activity. The FRAP values have been reported to range from 15 to 31 mM and from 2.2 to 5.5 mMFe_2_SO_4_ eq./L of red and white wine, respectively [53,54]. These values should be compared with those found in special beers (3.9–10.2 mM Fe_2_SO_4_ eq./L) and conventional beers (1.7–3.9 mM Fe_2_SO_4_ eq./L). Again, the antioxidant activity of special beers was comparable or even higher than that reported for white wines, although lower with respect to the antioxidant activity reported for red wines.

From our data, food addition during the fermentation step resulted in considerable improvement of the nutritional quality of beer, in terms of bioactive compounds content and antioxidant activity as compared with conventional beers. The increased amounts of polyphenols, particularly phenolic acids; flavonoids; and resveratrol in special beers have beneficial effects on beer drinkers.

Phenolic acids are small molecules with known antioxidant activity, acting as free radical acceptors and chain breakers. The antioxidant and biological effects, such as anti-inflammatory, cardioprotective, neuroprotective, antimicrobial, antiviral, anticancer effect of phenolic acids have been widely studied and reported in the literature, particularly for caffeic, ferulic, *p*-coumaric, and vanillic acids. Phenolic acids from beer have been described as being quickly absorbed and extensively metabolized in humans to the form of glucuronide and sulfate derivatives [55,56], which have been reported to retain antioxidant activity [57]. Flavonoids, the most abundant phenolic antioxidants in human diets, have been reported to be absorbed in humans, circulate in plasma, and are excreted in urine [2]. Flavonoids have been reported to display antioxidant activity, free radical scavenging capacity, metal chelation activity, coronary heart disease prevention, hepatoprotective, anti-inflammatory, and anticancer activities [49,58]. In regard to the stilbene resveratrol, bioavailability studies in humans have demonstrated its absorption and rapid metabolism to glucuronides and sulfates conjugates, the major plasma and urine metabolites [6]. Resveratrol has been reported to have several health-promoting effects in both animals and humans such as antioxidant, anti-inflammatory, antidiabetic, and antiproliferative properties [59,60].

A renewed interest has been focused on beer, due to its phenolic antioxidant component coupled with low ethanol content. Moderate beer drinking has been reported to increase plasma antioxidant and anticoagulant activities, to positively affect plasma lipid levels, and to exert protective effects on cardiovascular risk in humans [9,10,11,12,61]. Moreover, beer drinking seems to have no effect or even an inverse effect on total homocysteine concentration [62]. In conclusion, beer can contribute to the overall dietary intake of antioxidants and food addition to beer can significantly strengthen this contribution.

In addition to polyphenols, the barley, hops, and plant food contained other antioxidants, such as carotenoids, tocopherols, and ascorbic acid. All these compounds could contribute to some extent to the overall antioxidant activity of beers.

## 4. Materials and Methods

### 4.1. Materials

Caffeic acid, vanillic acid, sinapic acid, syringic acid, *p*-coumaric acid, ferulic acid, *o*-coumaric acid, chlorogenic acid (5-*O*-caffeoylquinic acid), catechin, epicatechin, resveratrol, myricetin, quercetin, trolox, gallic acid, ferric chloride, ferrous sulfate, sodium nitrite, aluminium chloride, potassium peroxodisulfate, 2,4,6-tripyridyl-S-triazine (TPTZ), 2,2′-azino-bis(3-ethylbenzothiazoline-6-sulfonic acid) diammonium salt (ABTS), and EDTA were from Sigma (St. Louis, MO, USA). Rutin was from Extrasynthese (Genay Cedex, France). Ascorbic acid and all organic solvents were obtained from Carlo Erba (Milano, Italy). Standard phenolics were dissolved in methanol (1 mg/mL), stored at −80 °C, and used within 1 week. Working standard solutions were obtained daily by dilution in sample buffer (1.25% glacial acetic acid, 7% methanol in twice-distilled water).

### 4.2. Beers

The conventional and special beers used in this study were purchased at local markets and beer shops. All special beers were produced by manufacturers with food addition during the first step of the fermentation process.

Special beers from the following different food typologies were explored: walnut (*Juglans regia* L. from Sorrento, Italy), chestnut (*Castanea Sativa* L. from Val Mongia, Italy), cocoa (*Theobroma Cacao* L.), honey (*Wildflower honey*), green tea (*Camelia Sinensis* L.), coffee (*Coffea Arabica L., Coffea Robusta* L.), and licorice (*Glycyrrhiza Glabra* L.). Table 1 showed the ingredients used for the beers’ production and the amount of foods added during the first fermentation step.

Beer bottles were stored in the dark and analyzed immediately after opening. Aliquots were frozen at −80 °C for phenolics profile determination and analyzed within one week.

### 4.3. Beers’ Analyses

The total polyphenols content was measured on 0.02 mL aliquots by the Folin–Ciocalteu method [63], using gallic acid as a reference compound. Briefly, beer samples were diluted with distilled water to give a final volume of 1 mL, then 0.1 mL of Folin–Ciocalteu’s reagent was added. After 5 min, 0.2 mL sodium carbonate (35% *w*/*v*) was added. Final volume was adjusted to 2 mL with distilled water. After 1 h in the dark, absorbance at 765 nm was measured against an appropriate blank reagent. The results were expressed as milligrams of gallic acid equivalents per liter of beer.

The total flavonoids content was measured on 0.05 mL aliquots by a colorimetric method previously described [64], using catechin as the reference standard to obtain the calibration curve. Briefly, beer samples were diluted with distilled water to a final volume of 1.5 mL, and then 0.075 mL of 5% NaNO_2_ solution was added. After 6 min, 0.15 mL of 10% AlCl_3_ hexahydrate was added and allow to stand for another 5 min, before 0.5 mL 1 M NaOH was added. The volume was adjusted to 2.5 mL with distilled water, mixed, and absorbance at 510 nm was measured. The results are expressed as milligrams of catechin equivalents per liter of beer.

The total antioxidant activity of beers was evaluated by both the ferric reducing antioxidant power (FRAP) assay [65] and by the ABTS radical cation decolorization (ABTS) assay [66] on 0.01 mL of beer aliquots. FRAP assay is a colorimetric method that measures the reduction of a ferric-tripyridyltriazine complex to its ferrous colored form, in the presence of antioxidants. The reaction was monitored for 6 min after the addition of beer to the FRAP reagent and the 6 min absorbance readings used for calculation referring to the iron sulfate calibration curve (range 0–100 μM) and reported as mM Fe_2_SO_4_ equivalent/L of beer. The ABTS assay is based on free radical scavenging capacity. The ABTS radical cation was produced by reacting ABTS solution (7 mM) with potassium persulfate (2.45 mM final concentration) in distilled water at room temperature, in the dark, for 16 h before use. A working solution (ABTS reagent) was diluted to obtain absorbance values between 1.4 and 1.5 AU at 734 nm and prewarmed at 30 °C. The percentage inhibition of absorbance was calculated with reference to a Trolox calibration curve (0–15 μM and expressed as mM Trolox equivalent/L of beer. All solutions were prepared daily.

International Bitterness Unit (IBU) and European Brewery Convention (EBC) values were supplied by the manufacturer. The IBU values measure the bitterness of beer, due to the amount of iso-alpha-acids. The EBC values refer to the color intensity, roughly darkness of the beer.

### 4.4. Beer Treatment for Phenolics Profile Determination by High Performance Liquid Chromatography (HPLC)

Beer aliquots (1 mL) were added with *o*-coumaric acid (10 μg) as internal standard and NaCl (300 mg). Phenolics were extracted with diethylether and diethylacetate, as described by Pozo-Bayon et al. [67]. Pooled extracts were evaporated under vacuum at 30 °C by rotatory evaporator. For total phenolic acids determination, beer samples were added with *o*-coumaric acid (20 μg) as internal standard and hydrolyze by alkaline treatment in the presence of ascorbate and EDTA [20,39]. After hydrolysis, the samples were acidified at pH 3.0 with 4 N HCl, added with NaCl (300 mg) and extracted as above reported. The dried residues, obtained by the above reported procedures, were dissolved in 0.1 mL methanol, vortexed for 5 min, and then EDTA (0.5 M, 40 μL), ascorbic acid (5% *w*/*v*, 0.2 mL), and twice-distilled water up to 1 mL final volume, were added. Samples were vortexed for 5 min, filtered, and analyzed by HPLC after appropriate dilution. Quantification of phenolic compounds was calculated with reference to calibration curves obtained with pure standard phenolics (range 0.1–10 μg injected).

Recovery experiments were performed adding known amounts of pure phenolic compounds to beer samples, followed by the above reported extraction protocol. An almost complete recovery of all phenolics under study was measured (range 91.0%–105.8%). When samples were submitted to alkaline hydrolysis, prior to the extraction procedure for the total phenolic acids evaluation, the recovery of the phenolic acids under study was in the range 95.9%–104.6%.

### 4.5. HPLC Instrumentation

In our laboratory, phenolic compounds are routinely assayed in foods, beverages, human plasma, and cell extracts using high performance liquid chromatography (HPLC) [13,16,39,53,68]. The high performance liquid chromatograph is a PerkinElmer Series 200 Liquid Chromatography (PerkinElmer, Norwalk, CT, USA) with gradient pump, column thermoregulator, auto-sampling injector, and diode array detector (DAD) (PerkinElmer Norwalk, CT, USA). The operating conditions used were as follows: column temperature 30 °C, flow rate 1 mL/min, injection volume 50 μL, and detector at 280 nm. Chromatographic separation was obtained on a Supelcosil LC-18 column (5.0 μm particle size, 250 × 4.6 mm ID), equipped with a guard column (C_18_, 5.0 μm particle size, 20 × 4.0 mm ID; both Supelco, Bellefonte, PA, USA).To separate phenolic compounds, a gradient elution was performed using the following two mobile phases: solution A, consisting of 1.25% glacial acetic acid in twice-distilled water and solution B, absolute methanol. The gradient used was as follows: 0–30 min, from 98% A, 2% B to 94% A, 6% B, linear gradient; 31–60 min, from 94% A, 6% B to 88% A, 12% B, linear gradient; 61–80 min, from 88% A, 12% B to 74% A, 26% B, linear gradient; 81–95 min, from 74% A, 26% B to 65% A, 35% B, linear gradient; 96–105 min, from 65% A, 35% B to 60% A, 40% B, linear gradient; and 106–120 min, 45% A, 55% B; 121–150 min, 98% A, 2% B.

### 4.6. Statistical Analysis

Data presented are means ± standard error. All measurements were made at least in triplicate. Statistical analysis was performed using a statistical package running on a PC (KaleidaGraph 4.0, Synergy Software, Reading, PA, USA). The Student’s *t* test was used for regression analyses. The probability of *p* < 0.05 was considered to be statistically significant.

## Figures and Tables

**Table 1:** Ingredients of the special and conventional beers.

Beer Code	Food Added	Amount Added (g/L of Beer)	Ingredients
**Special Beers:**			
**WALN**	Walnut	35	Water, barley malt, oats, walnut, hops, yeast
**CHES**	Chestnut	40	Water, barley malt, dried chestnut, hops, yeast
**GTEA**	Green tea	9	Water, barley malt, wheat malt, hops, yeast, green tea
**COFF**	Coffee	35	Water, barley malt, oats, coffee (80% Arabica, 20% Robusta), hops, yeast
**COCO**	Cocoa beans	10	Water, barley malt, oats, carob, cocoa beans, hops, yeast
**HONE**	Honey	62	Water, barley malt, wildflower honey, hops, yeast
**LIQU** **Conventional Beers:**	Licorice	2	Water, barley malt, wheat malt, hops, licorice, sugar, yeast
**ALE 1**	-	-	Water, barley malt, corn, barley, hops, yeast
**ALE 2**	-	-	Water, barley malt, sugar, hops, yeast
**ALE 3**	-	-	Water, barley malt, caramelized barley malt, hops, yeast
**LAGE 1**	-	-	Water, barley malt, maize, hops, yeast
**LAGE 2**	-	-	Water, barley malt, barley, glucose syrup, hops, yeast

**Table 2:** Characteristics, bitterness, pH, and color measurements of special and conventional beers.

Beer Code	Style	Country of Production	Alcohol Strength (% vol)	pH ^a^	IBU	Color EBC
**Special Beers:**						
WALN	Ale	Italy	4.7	4.47	30	87
CHES	Lager	Italy	8.0	4.64	7	61
GTEA	Ale	Italy	4.5	4.54	12	5
COFF	Ale	Italy	4.5	4.04	15	110
COCO	Ale	Italy	7.0	4.41	10	110
HONE	Ale	Italy	6.8	4.34	8	18
LIQU	Ale	Italy	9.0	4.60	22	70
**Conventional Beers:**						
ALE 1	Ale	Belgium	6.6	4.39	28	15
ALE 2	Ale	Italy	5.2	4.61	25	8
ALE 3	Ale	Italy	5.2	4.29	35	20
LAGE 1	Lager	Italy	4.6	4.43	15	7
LAGE 2	Lager	Italy	4.8	4.87	20	4

Alcohol strength, IBU, and EBC values were provided by manufacturers. ^a^ Values are mean of three independent experiments. Standard error was < 0.02.

**Table 3:** Antioxidant activity, total polyphenols and total flavonoids contents of special and conventional beers.

Beer Code	Total Polyphenols *Gallic acid Eq.* *mg/L*	Total Flavonoids *Catechin Eq.* *mg/L*	FRAP *Fe_2_SO_4_ Eq.* *mM*	ABTS *Trolox Eq.* *mM*
***Special Beers:***				
**WALN**	964.7 ± 9.6 ^a^	90.1 ± 1.8 ^a^	10.2 ± 0.02 ^a^	5.2 ± 0.05 ^a^
**CHES**	883.4 ± 10.9 ^b^	71.7 ± 0.9 ^b^	6.2 ± 0.08 ^b^	3.4 ± 0.03 ^b^
**GTEA**	464.4 ± 3.9 ^f^	42.0 ± 0.3 ^e^	3.6 ± 0.05 ^d^	2.4 ± 0.03 ^e^
**COFF**	582.7 ± 6.4 ^d^	69.5 ± 1.0 ^b^	5.0 ± 0.14 ^e^	2.9 ± 0.03 ^f^
**COCO**	1026.4 ± 3.0 ^a^	96.4 ± 2.0 ^c^	8.1 ± 0.10 ^c^	3.9 ± 0.04 ^c^
**HONE**	538.3 ± 8.3 ^e^	48.7 ± 1.0 ^f^	3.9 ± 0.01 ^f^	2.5 ± 0.03 ^d^
**LIQU**	819.7 ± 6.9 ^c^	81.4 ± 1.3 ^d^	6.1 ± 0.04 ^b^	3.4 ± 0.01 ^b^
***Conventional Beers:***				
**Ale 1**	446.1 ± 12.6 ^f,i^	51.9 ± 1.1 ^g^	3.7 ± 0.17 ^d,f,h^	1.7 ± 0.03 ^g,h^
**Ale 2**	382.7 ± 6.6 ^l^	59.0 ± 0.9 ^h^	3.4 ± 0.04 ^h^	1.5 ± 0.02 ^i^
**Ale 3**	424.4 ± 8.7 ^g,f^	51.9 ± 1.3 ^f,g^	3.9 ± 0.01 ^f^	2.6 ± 0.02 ^d^
**LAGE 1**	273.8 ± 4.1 ^h^	26.6 ± 0.1 ^l^	1.7 ± 0.02 ^g^	1.8 ± 0.03 ^g^
**LAGE 2**	320.6 ± 8.6 ^m^	63.5 ± 0.8 ^i^	2.8 ± 0.04 ^i^	1.5 ± 0.06 ^h,i^

FRAP, ferric reducing antioxidant power assay; ABTS, 2,2′-azino-bis (3-ethylbenzothiazoline-6-sulfonic acid) assay. Values are means ± SE (polyphenols content, n = 5; flavonoids content, n = 6; FRAP and ABTS, n = 3). Within each column, values with different superscript are significantly different (*p* < 0.05, one-way ANOVA, Fisher method).

**Table 4:** Phenolic acids, flavonoids, and resveratrol contents of conventional beers by high performance liquid chromatography with diode array detector (HPLC-DAD) (mg/L).

Beer Code	ALE 1	ALE 2	ALE 3	LAGE 1	LAGE 2
***Phenolic Acids:***					
**Chlorogenic**	nd	nd	nd	nd	nd
**Vanillic**					
*Free*	nd	nd	2.09 ± 0.08	nd	nd
*Total*	2.80 ± 0.05	3.58 ± 0.07	4.65 ± 0.06	4.46 ± 0.12	2.3 ± 0.07
**Caffeic**					
*Free*	nd	nd	1.24 ± 0.10	nd	nd
*Total*	3.00 ± 0.20	3.38 ± 0.01	5.99 ± 0.11	1.70 ± 0.08	1.61 ± 0.04
**Syringic**					
*Free*	nd	nd	0.25 ± 0.01	nd	nd
*Total*	0.71 ± 0.09	0.67 ± 0.04	0.51 ± 0.03	nd	0.32 ± 0.01
***p*-Coumaric**					
*Free*	0.53 ± 0.03	1.12 ± 0.05	0.68 ± 0.02	1.06 ± 0.05	0.35 ± 0.04
*Total*	2.00 ± 0.10	2.77 ± 0.09	2.13 ± 0.04	1.56 ± 0.08	0.77 ± 0.01
**Ferulic**					
*Free*	0.90 ± 0.03	11.03 ± 0.54	2.91 ± 0.11	2.12 ± 0.06	1.81 ± 0.03
*Total*	10.27 ± 1.00	19.90 ± 0.21	21.66 ± 0.55	11.0 ± 0.07	13.71 ± 0.49
**Sinapic**					
*Free*	0.41 ± 0.01	0.44 ± 0.21	0.98 ± 0.10	0.36 ± 0.04	1.07 ± 0.04
*Total*	4.80 ± 0.05	2.19 ± 0.07	3.95 ± 0.11	3.53 ± 0.03	3.07 ± 0.06
***Total Phenolic Acids ^a^***	23.58 ± 1.56	32.49 ± 0.49	38.89 ± 0.90	22.25 ± 0.38	21.78 ± 0.68
***Flavonoids:***					
**Catechin**	nd	nd	nd	nd	nd
**Epicatechin**	nd	nd	nd	nd	nd
**Rutin**	nd	nd	nd	nd	nd
**Myricetin**	nd	nd	nd	nd	nd
**Quercetin**	nd	nd	nd	nd	nd
***Stilbenes:***					
**Resveratrol**	nd	nd	nd	nd	nd

Values are means ± SE (n = 3). nd, not detectable. ^a^ Total phenolic acids content was calculated by the sum of single phenolic acids content obtained after alkaline hydrolysis.

**Table 5:** Phenolic acids, flavonoids, and resveratrol contents of special beers by HPLC-DAD (mg/L).

Beer Code	WALN	CHES	GTEA	COFF	COCO	HONE	LIQU
***Phenolic Acids:***							
**Chlorogenic**	tr	nd	nd	1.56 ± 0.10	nd	nd	nd
**Vanillic**							
*Free*	0.92 ± 0.12	1.57 ± 0.03	0.87 ± 0.04	0.78 ± 0.03	1.14 ± 0.09	0.80 ± 0.05	1.03 ± 0.10
*Total*	2.16 ± 0.26	5.09 ± 0.06	2.82 ± 0.15	2.03 ± 0.14	3.39 ± 0.17	3.09 ± 0.22	2.32 ± 0.11
**Caffeic**							
*Free*	0.52 ± 0.01	0.24 ± 0.01	tr	0.57 ± 0.02	0.50 ± 0.02	tr	0.56 ± 0.07
*Total*	3.16 ± 0.15	3.47 ± 0.03	1.48 ± 0.18	9.20 ± 0.21	3.69 ± 0.01	2.37 ± 0.17	3.71 ± 0.04
**Syringic**							
*Free*	tr	0.40 ± 0.03	0.62 ± 0.02	tr	0.54 ± 0.02	0.27 ± 0.01	0.40 ± 0.03
*Total*	tr	1.24 ± 0.05	0.96 ± 0.04	tr	1.42 ± 0.05	1.24 ± 0.10	0.67 ± 0.03
***p*-Coumaric**							
*Free*	0.68 ± 0.01	1.02 ± 0.06	0.11 ± 0.01	0.36 ± 0.02	1.38 ± 0.08	0.21 ± 0.01	1.06 ± 0.06
*Total*	4.32 ± 0.24	3.36 ± 0.07	2.24 ± 0.16	1.93 ± 0.08	3.26 ± 0.13	1.75 ± 0.03	2.95 ± 0.14
**Ferulic**							
*Free*	1.05 ± 0.02	1.81 ± 0.20	0.15 ± 0.01	0.63 ± 0.02	1.16 ± 0.03	0.43 ± 0.01	1.32 ± 0.08
*Total*	8.22 ± 0.17	27.55 ± 0.43	14.30 ± 0.40	20.50 ± 0.64	22.10 ± 0.73	19.20 ± 0.33	20.63 ± 0.87
**Sinapic**							
*Free*	0.45 ± 0.01	0.97 ± 0.12	0.49 ± 0.01	0.24 ± 0.02	0.44 ± 0.01	0.55 ± 0.01	1.03 ± 0.04
*Total*	2.68 ± 0.06	4.74 ± 0.04	4.48 ± 0.08	2.52 ± 0.02	4.89 ± 0.05	6.73 ± 0.03	6.66 ± 0.07
***Totalphenolic Acids ^a^***	20.54 ± 0.88	45.45 ± 0.68	26.28 ± 1.01	36.18 ± 1.09	38.75 ± 1.14	34.38 ± 0.88	36.94 ± 1.26
***Flavonoids:***							
**Catechin**	tr	4.65 ± 0.13	2.98 ± 0.09	tr	4.58 ± 0.02	tr	tr
**Epicatechin**	1.80 ± 0.11	3.68 ± 0.12	3.09 ± 0.05	1.30 ± 0.07	1.83 ± 0.11	0.94 ± 0.05	tr
**Rutin**	nd	nd	0.68 ± 0.02	nd	nd	1.29 ± 0.02	0.92 ± 0.10
**Myricetin**	4.44 ± 0.27	tr	1.69 ± 0.05	0.39 ± 0.03	0.65 ± 0.02	2.67 ± 0.18	8.82 ± 0.07
**Quercetin**	6.55 ± 0.31	tr	1.17 ± 0.09	0.54 ± 0.02	1.52 ± 0.06	4.67 ± 0.23	2.63 ± 0.15
***Stilbenes:***							
**Resveratrol**	0.26 ± 0.20	0.35 ± 0.02	0.32 ± 0.02	0.23 ± 0.01	0.31 ± 0.01	0.24 ± 0.01	0.20 ± 0.01

Values are means ± SE (*n* = 3). Nd, not detectable and tr, traces amount.^a^ Total phenolic acids content was calculated by the sum of single phenolic acids content obtained after alkaline hydrolysis.
